# The IL-17 pathway as a target in giant cell arteritis

**DOI:** 10.3389/fimmu.2023.1199059

**Published:** 2024-01-17

**Authors:** Markus Zeisbrich, Jens Thiel, Nils Venhoff

**Affiliations:** ^1^ Department of Rheumatology and Clinical Immunology, Medical Center – University of Freiburg, Freiburg, Germany; ^2^ Division of Rheumatology and Clinical Immunology, Medical University Graz, Graz, Austria

**Keywords:** vasculitis, giant cell arteriitis (GCA), IL-17, secukinumab, Th-17

## Abstract

The network of IL-17 cytokines is considered a key component of autoimmune and inflammatory processes. Blocking IL-17 showed great success in psoriasis as well as psoriatic arthritis, and in patients with axial spondyloarthritis. Secukinumab is one of the approved IL-17A inhibitors for these diseases and is now routinely used. In giant cell arteritis, a large vessel vasculitis, there is accumulating evidence for a pathogenic role of IL-17 and Th17 cells, which are part of the CD4^+^ T-cell subset. Giant cell arteritis occurs in individuals over 50 years of age and many have relative contraindications to glucocorticoid therapy, which today still represents the mainstay therapy. Despite the approval of tocilizumab, which targets the IL-6 receptor, a high demand for glucocorticoid-sparing agents remains that combine the effective suppression of the acute inflammation observed in giant cell arteritis with a safety profile that matches the needs of an older patient population. The first results from a phase II proof-of-principle study (TitAIN) support an optimistic outlook on a potential new treatment option with secukinumab in giant cell arteritis.

## Introduction

Interleukin (IL)-17 was discovered 30 years ago (1, 2) and is now recognized as one of the main cytokines for barrier protection on epithelial and mucosal surfaces with an essential function in immunity to mucosal fungal infections (3). Moreover, its pro-inflammatory function is relevant in immune-mediated diseases, first demonstrated in a mouse model for multiple sclerosis (4). The identification of the immuno-pathological role of IL-17 subsequently led to the development of IL-17 blocking agents. While the use of monoclonal anti-IL-17A antibodies was disappointing in rheumatoid arthritis and multiple sclerosis (5, 6), these therapeutics are now used with great success in patients with psoriasis, psoriatic arthritis, and axial spondyloarthritis (7, 8).

Giant cell arteritis (GCA) leads to vascular inflammation in large arteries as well as medium-sized arteries with a tissue tropism preferentially affecting the thoracic aorta, its proximal branches (e.g., A. subclavia, A. carotidea, and A. axillaris), and cranial arteries (e.g., A. temporalis superficiales and occipital arteries) (9). Vascular inflammation drives circular vessel wall thickening and luminal occlusion, which may cause ischemic symptoms like headaches, visual symptoms or blindness, scalp tenderness, or jaw claudication when branches of the carotid arteries are affected (10). GCA generally occurs over 50 years of age with the highest incidence among persons who are 75 to 85 years old (11). Glucocorticoids are still the standard treatment although their long-term use entails grave side effects in this older patient population (12, 13).

This review focuses on IL-17 and Th17 cells, and presents available evidence for the involvement of the IL-17 pathway in GCA. Additionally, the results of the phase II proof-of-concept study TitAIN with an IL-17A blocking agent in GCA patients with active disease are depicted and discussed.

## Signaling and effector functions of the IL-17 cytokine family members

Six isoforms of IL-17 (IL-17A to IL-17F) are identified with IL-17A and IL-17F having the greatest homology and some overlapping functions (14, 15). IL-17A is the most intensively studied isoform and more potent than its closest sibling IL-17F (16). IL-17A and IL-17F can exist as homodimers or form IL-17A/F heterodimers and they are co-expressed by linked genes (17). Monoclonal antibodies such as secukinumab and ixekizumab directly bind IL-17A, while bimekizumab neutralizes both IL-17A and IL-17F.

IL-17 signaling is mediated through five receptors (IL-17RA to IL-17RE) (18). The IL-17 receptors RA, RC, and RD are required for the signaling of IL-17A and IL-17F (19). Receptor ligation activates common downstream intracellular signaling via nuclear factor-kappa B (NF-κB), mitogen-activated protein kinases (MAPK), CCAAT/enhancer-binding protein (C/EBP), Janus kinase (JAK), PI3K (phosphatidylinositol 3-kinase), and the JAK/STAT pathway (20) to promote gene transcription. In homeostasis, large numbers of IL-17-producing cells reside on mucocutaneus surfaces (19) and IL-17 is a key cytokine in promoting barrier functions in order to limit fungal and bacterial invasion (3). Defects in IL-17 production lead to impaired protection against candida and other fungal pathogens (21, 22). Thus, patients under treatment with anti-IL-17 mAbs are at risk to suffer from mostly mild and localized mucocutaneous candidiasis (23).

Another important aspect of IL-17-mediated host protection is executed by the recruitment of neutrophils. IL-17 increases the release of IL-8 (also called CXCL8 or neutrophil chemotactic factor) in target cells (24), which is a chemokine-inducing chemotaxis of neutrophils towards the side of its production, and the production of G-CSF, a major cytokine for proliferation, differentiation, and function of neutrophils (25, 26).

Another characteristic of IL-17 is its cooperative effect with other inflammatory mediators. Not only do the two isoforms IL-17A and IL-17F act synergistically with each other (27), but also other cytokines like granulocyte-macrophage colony-stimulating factor (GM-CSF), IFN-, IL-22, IL-1β, and TNF-α cooperate with IL-17 to exert pro-inflammatory effects (28). The synergy between IL-17 and TNF-α becomes evident on the post-transcriptional level. IL-17 or TNF-α alone are both rather weak inducers of messenger RNA for CXCL1, CXCL2, IL-6, I-κBζ, and CXCL5, which is then subject to rapid degradation. In contrast, when IL-17 and TNF-α act synergistically, the stability of those messenger RNAs is enhanced significantly (29). In essence, despite being a faint inducer of target genes, synergistic effects of IL-17 together with other cytokines potently shape inflammatory gene expression patterns.

In 2005, CD4^+^ T helper 17 (Th17) cells were described as the major source of IL-17 (30, 31). Since then, many other cell types with the ability to produce IL-17 have been identified: innate lymphoid cells and natural killer cells, γδ T cells and CD8+ T cells, NK T cells and mucosal-associated T cells, Paneth cells, and mast cells (3, 32–35). Owing to their relevance in GCA, this mini-review focuses on Th17 cells.

## The differentiation and the developmental pathway of Th17 cells

The CD4^+^ T-cell lineage is considered to have four major subsets with Th1 cells, Th2 cells, Th17 cells, and T regulatory cells (Tregs). Of those, Th17 cells characteristically secrete high levels of IL-17A, IL-17F, and also IL-22 and GM-CSF. Interestingly, *in vitro* differentiated Th17 cells produce more IL-17F than IL-17A, while the majority of pathogenic Th17 cells in inflammatory diseases produce both cytokines at a similar level (36).

Differentiation of Th17 cells is a rather complex process compared to differentiation of other cellular lineages, e.g., Th1 or Th2 cells. Induction of Th17 cells is orchestrated by multiple cytokines with transforming growth factor-beta (TGF-β) and IL-6 being the primary cytokines initiating Th17 differentiation (37). In addition, co-signaling with IL-1β and IL-23 further guides Th17 survival, proliferation, and development (38, 39). On the transcriptional level, RORγt and STAT3 cooperatively induce expression of IL-17. RORγt is acknowledged as the key transcription factor in this process; its overexpression alone induces *IL17* transcription in the absence of other cytokines, while STAT3 overexpression fails to do so in the absence of RORγt (40).

The developmental pathway of Th17 cells is interconnected with that of Tregs as TGF-β is crucial for both subsets to induce differentiation. Adding IL-6 then shifts CD4^+^ T cells towards the Th17 lineage by inhibiting FoxP3, the key transcription factor of Tregs, and upregulating RORγt (41, 42). The association of a reduced expression of FoxP3 in T cells together with the loss of immunological tolerance and autoimmunity is reported repeatedly (43–45). Thus, the homeostasis between Th17 cells on one side and Tregs on the other side is an important gatekeeper in the prevention of uncontrolled inflammation and autoimmunity (46).

Because of their plasticity, the phenotypical fate of Th17 cells and Tregs depends on the stimulation they receive from their immunological microenvironment. In this context, cytokines from innate immune cells are particularly able to push these cells into a pro-inflammatory and pathogenic direction. IL-6 inhibits the key transcription factor of Tregs while, at the same time, enhancing the key transcription factor of Th17 cells. IL-1β boosts Th17 cell differentiation, promotes expression of IL-17, and suppresses the production of anti-inflammatory IL-10 in Th17 cells (47), enhancing their pathogenic potential in autoimmune disease (48). IL-23 is required for the stabilization and expansion of the Th17 lineage (39). Therefore, these cytokines are sometimes called Th17-related cytokines. On the other hand, IL-2 is indispensable for the differentiation and proliferation of Tregs (49) and low-dose IL-2 enables a shift from the Th17 population toward Treg cells (50).

## Evidence for the involvement of the IL-17 pathway in giant cell arteritis

In 2008, an *in vivo* model by Chen et al. (51) studied the impact of interferon regulatory factor 4 (IRF-4) and IRF-4-binding protein (IBP) on the Th17 lineage. Transgenic IBP-deficient mice were characterized by hyperresponsive CD4+ T cells with increased production of IL-17 and IL-21, both signature cytokines of Th17 cells. To the investigators’ surprise, IBP-deficient mice started dying from an age of 3 months or older and histologic analysis revealed that all mice suffered from severe inflammation of the aortic roof resembling granulomatous large vessel vasculitis with the presence of multinucleated giant cells, a typical finding in GCA. This is the first *in vivo* model pointing to a role of IL-17 in large vessel vasculitis.

In GCA, increased production of IL-6 was first reported in 1993 by Roche et al. (52). Based on findings on the Th17 lineage published almost 20 years later, one could conclude that IL-6 tips the balance from Tregs towards Th17 cells in vasculitis patients. Indeed, in 2012, two independent studies reported an expansion of the Th17 population in the peripheral blood of GCA patients while Treg counts were decreased at the same time (53, 54).

Another study showed Treg dysfunction in GCA patients and downregulation of FoxP3 in GCA Tregs. Interestingly, treatment with tocilizumab partially normalized Treg dysfunction but not FoxP3 expression (55). Miyabe et al. demonstrate that GCA Tregs from active disease possessed impaired suppressive capacity and a hypofunctional isoform of FoxP3 with missing exon 2. These cells displayed a Th17-like phenotype, which, again, illustrates the plasticity of Tregs. In this study, tocilizumab but not corticosteroids were able to correct Treg abnormalities (56).

An important study by Deng et al. illustrated that in patients with active GCA, the subsets of Th1 and Th17 cells were both expanded, but only the presence of relevant Th17 cell numbers was characteristic of new-onset disease. While Th1 cells already accounted for 11.8% of CD4 T cells in age-matched healthy controls with an increase to 20.6% in GCA patients, Th17 cells in controls were only marginal with 0.03% to 0.59% and increased in GCA patients 8-fold, making up to 5.3% of CD4 T cells in individual cases. In temporal artery biopsies, IL-17-producing cells were abundant in all wall layers and around adventitial vasa vasorum. This study also delivered evidence that the underlying immune defect might emerge from the innate immune system as monocytes from GCA patients were capable of inducing Th17 differentiation by producing IL-1β, IL-6, and IL-23. Glucocorticoid treatment normalized Th17 cell counts within 10 weeks, while Th1 cell frequency was unaffected (57).

In line with this, patients with high IL-17A expression in temporal artery specimens had fewer relapses and required shorter duration of glucocorticoid treatment in another study (58), indicating that the IL-17 pathway is involved in the acute phase of the disease and is suppressed by glucocorticoids.

Work from Wen et al. shed light on the interaction of the GCA vasculature with T cells. Adventitial microvascular endothelial cells control the access of inflammatory cells to the inner vascular wall, and infiltration of immune cells from the adventitia to the media and intima is a key step in the pathophysiology of GCA. High levels of vascular endothelial growth factor (VEGF) in GCA blood induce the expression of the Notch ligand Jagged1 on adventitial endothelial cells. This induces differentiation of CD4^+^ T that express the Notch1 receptor towards pathogenic Th1 and Th17 cells (59).

Investigating the histological pattern in GCA-affected arteries, Ciccia et al. reported IL-17 expression mainly in arteries displaying granulomatous transmural inflammation—with IL-17-positive giant cells—and vasa vasorum vasculitis. Furthermore, the expression of IL-17 in arteries was directly correlated with the intensity of the systemic inflammatory response in GCA patients (60). To expand the complex histological picture of vasculitis in GCA, a recent study reported neutrophil extracellular traps (NETs) in temporal arteries from patients with GCA—found primarily in the adventitia close to vasa vasorum. In all of the 10 patients, these NETs were decorated with IL-17A (61).

Epigenetic investigations of temporal arteries revealed hypomethylated genes that indicate strong activation of Th1 and Th17 pathways (62). Last, a meta-analysis including over 1,000 GCA patients and more than 3,000 healthy individuals from Spain, Italy, Germany, and Norway revealed that polymorphisms (SNPs rs4711998, rs2275913, and rs7747909) within the IL-17A locus confer a risk to GCA (63).

## IL-17A inhibition in giant cell arteritis

The first case report of successful GCA treatment with a monoclonal antibody against IL-17A (secukinumab) appeared in 2018 in a patient with additional psoriatic arthritis, in which secukinumab is approved for therapy (64). In addition to psoriatic arthritis, secukinumab is approved for the treatment of plaque psoriasis and active non-radiographic and radiographic axial spondyloarthritis (65).

The phase II proof-of-concept trial TitAIN (66) was the first randomized controlled trial investigating the efficacy and safety of secukinumab in patients with active GCA. This randomized, parallel-grouped, double-blinded, placebo-controlled multi-center study was designed to compare secukinumab 300 mg to placebo in patients with active GCA with a 26-week prednisolone taper regimen (67). Fifty-two patients with relapsing (19.2%) or newly diagnosed (80.8%) GCA were included in the 52-week trial (66). A total of 37 patients (71.2%) completed the study and the proportion of GCA patients in sustained remission at week 28 was higher with secukinumab (70.1%) than with placebo (20.3%). At the end of the study in week 52, these numbers were 59.3% in the secukinumab arm and 8.0% in the placebo arm. In addition, secukinumab treatment led to a longer time to first GCA relapse compared to placebo treatment. Importantly, no unexpected or new safety signals compared to the use of secukinumab in other indications were detected (66).

For this reason, two phase III studies (NCT04930094 and NCT05380453) are currently recruiting to comprehensively investigate secukinumab in the treatment of active relapsing or newly diagnosed GCA. In addition, the treatment of patients with active polymyalgia rheumatica (PMR) without concomitant GCA is being investigated in a phase III study (NCT05767034).

## Discussion

**Figure 1:**
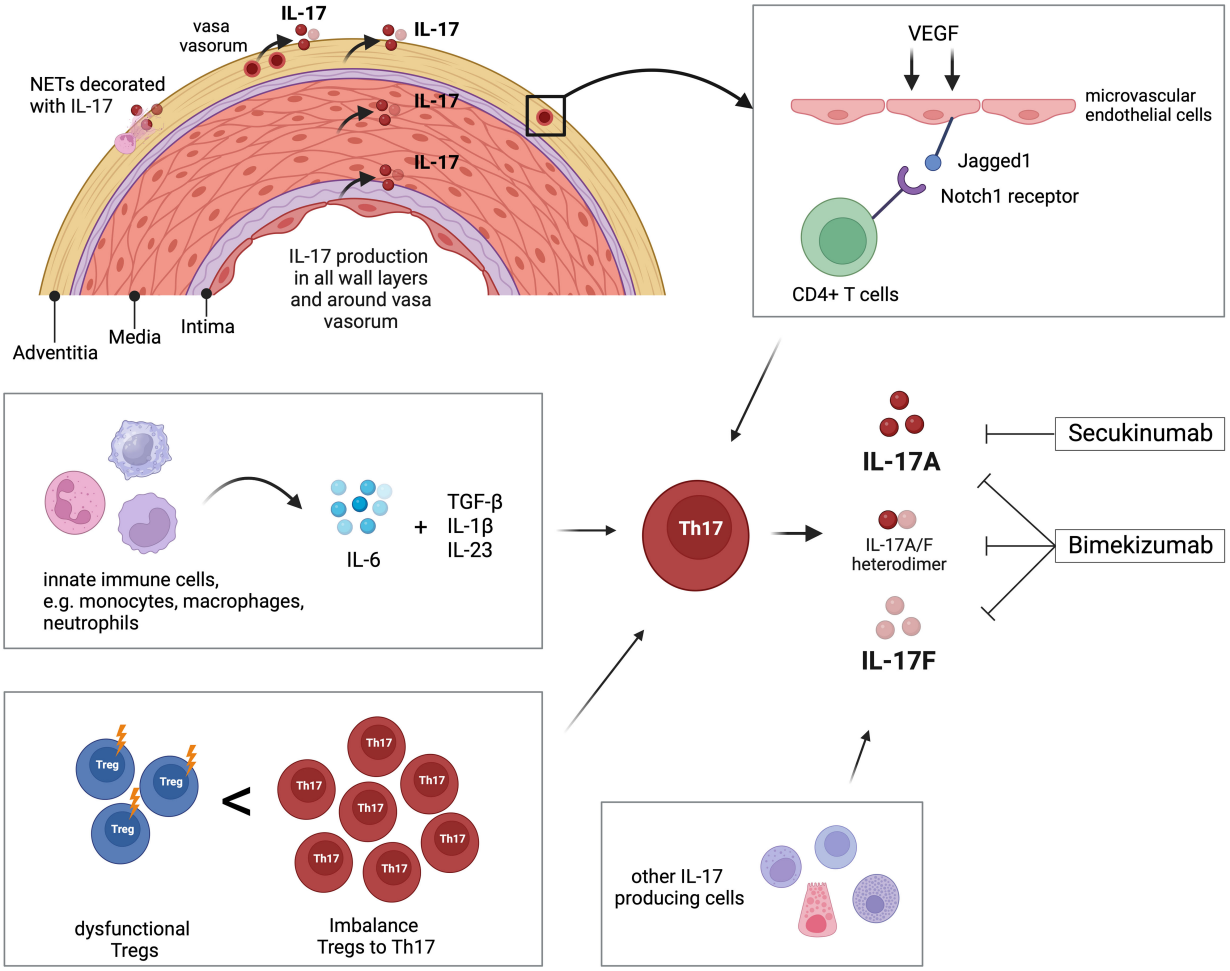
Overview of the role of IL-17 and Th17 cells in giant cell arteritis (GCA). Interleukin-17 (IL-17) is detectable in all layers of temporal arteries, around adventitial vasa vasorum, and in neutrophil extracellular traps (NETs) located in the adventitia. Microvascular endothelial cells in the adventitia are stimulated by vascular endothelial growth factor (VEGF) to present the Notch-ligand Jagged1 to bind CD4+ T cells, which then differentiate into Th17 cells. Innate immune cells like monocytes, macrophages, and neutrophils produce IL-6, which, in combination with TGF-β, IL-1β, and IL23, promotes differentiation of Th17 cells. The peripheral blood of GCA patients is characterized by increased frequency of Th17 compared to decreased number of T regulatory cells (Tregs). Additionally, those Tregs are dysfunctional regarding their regulatory function. Th17 cells and other immune and non-immune cells produce IL-17 that is linked to the pathology of GCA. The pharmaceutical agent secukinumab blocks IL-17A, while bimekizumab blocks IL-17A and IL-17F. Created with BioRender.com.

There is robust evidence for the involvement of the IL-17 pathway in the disease process of GCA (**Table 1**). Th17 cell populations are expanded in active GCA and IL-17-producing cells are abundant in all vascular wall layers of affected temporal artery specimens. As the developmental pathway of Th17 cells is interconnected with that of Tregs, it is interesting that, at the same time, Treg counts are lower in GCA patients and that these Tregs are dysfunctional. This leads to the suggestion that the dysbalance between these important T-cell subtypes is of relevance in the vasculitic process in GCA (**Figure 1**).

**Table 1:** Supporting findings for the contribution of IL-17 to the pathogenesis of GCA.

Finding	Ref.
In transgenic mice, hyperresponsive CD4^+^ T cells with enhanced IL-17 production associated with sudden onset of large vessel vasculitis	(51)
Expansion of Th17 population in peripheral blood of GCA patients while Treg counts are decreased	(53, 54)
Monocytes from GCA patients drive Th17 differentiation by producing IL-1b, IL-6, and IL-23	(57)
Glucocorticoid treatment normalized increased Th17 cell counts in GCA patients within 10 weeks, while Th1 cell frequency is unaffected	(57)
Adventitial microvascular endothelial cells instruct CD4^+^ T cells to differentiate into Th17 effector cells by Jagged1–Notch interaction	(59)
IL-17-producing cells are abundant in temporal artery specimens from GCA patients in all wall layers and are found around adventitial vasa vasorum	(57)
Patients with high IL-17A expression in temporal artery specimens have fewer relapses and require shorter glucocorticoid treatment periods	(58)
IL-17 expression in GCA arteries is directly correlated with the intensity of the systemic inflammatory response	(60)
Neutrophil extracellular traps located mainly in the adventitia are decorated with IL-17A	(61)
Polymorphisms (SNPs rs4711998, rs2275913, and rs7747909) within the IL-17A locus confer a risk to GCA	(63)
In the TitAIN study, newly diagnosed or relapsing active GCA is successfully treated by blockade of IL-17A	(66)

In contrast to many other rheumatic diseases, in which new therapies have emerged over the last decades, the standard of care in GCA is still defined by long-term treatment with high-dose glucocorticoids. Although this treatment approach effectively reduces systemic inflammation, histopathological studies of temporal artery biopsies to multiple time points in steroid-treated patients show that clinical symptoms were suppressed, but inflammatory alterations in the vascular wall often persisted (68). Furthermore, treatment with glucocorticoids entails high numbers of serious adverse effects (12, 69), which is especially relevant in GCA patients. They are, by definition, of older age, and many have relative contraindications to glucocorticoid therapy, e.g., diabetes or osteoporosis. This problem gets amplified by long-term intake of glucocorticoids when prednisolone cannot be reduced on time. In this context, another study showed that after 2 years, only 55% of patients have a daily dose of prednisolone lower than 5 mg (70). The only glucocorticoid-sparing agent approved for GCA so far is the IL-6 receptor antagonist tocilizumab. Yet, tocilizumab suppresses acute-phase proteins that are routinely used for GCA relapse detection, and MRI studies in tocilizumab-treated patients showed normalization of vessel wall enhancement only in one-third of the patients (71), leaving the remaining question of whether these findings have an impact on long-term prognosis.

In other words, there is a high need for other glucocorticoid-sparing agents for the treatment of GCA that combine effective suppression of acute inflammation with a safety profile that matches the needs of an elderly patient population. Considering these expectations, it was timely to investigate a pharmaceutical agent in GCA patients that has already been shown to be effective and safe in other rheumatic diseases. Secukinumab fulfilled these requirements and first results from the TitAIN study led to an optimistic outlook on a new potential treatment option in GCA.

## Author contributions

MZ, JT, and NV wrote the manuscript. All authors contributed to the article and approved the submitted version.
